# A Practical Treatise on the New Operation for Lateral Curvature of the Spine

**Published:** 1842-01

**Authors:** 


Art. IX.—*A Practical Treatise on the New Operation for Lateral Curvature of the Spine; showing those cases in which the operation is admissible. With Plates.* By G. B. Childs, m.r.c.s. &c. — *London*, 1841. 8vo, pp. 56.

The work before us bears no internal evidence whatever of any necessity or good reason for its appearance in the literary world, at least as *a book;* a page and a half of any journal being amply sufficient to contain the "kernel" that is housed in the fifty-six pages of husk and shell. We shall not, therefore, attempt any deliberate analysis of its contents, the pith of which may be given as follows:

The cases in which "the operation is alone admissible" are arranged under two heads: 1, "Those which are primarily and fundamentally dependent on increased muscular development;" 2, "Those in which the muscles are but passive agents in retaining the spine in its abnormal situation." (p. 7.) The first class of curvature is usually seated in the upper region of the back; contrary to the opinions of Messrs. Shaw, Skey, &c., it is usually the original curve, and when the lumbar region bends it does so secondarily; the latissimus dorsi, trapezius, and rhomboid muscles are those usually in fault; the right side is the one usually affected; the remote cause is peculiar manual avocation inducing excessive action of these muscles over that of their fellows; and the remedy consists in subintegumental division of the aforesaid muscles, close to the spine. The second class of curvature is usually seated in the lower parts of the spine; the longissimus dorsi and sacrolumbalis (which the author affectedly terms "sacrocostalis") are the offending muscles; they will be found constituting a tight faulty cord on one or other side of the curve, usually the convex, which cord is to be subintegumentally divided, and more especially its tendinous investing fascia; but on the whole, the author's views on this latter division of his subject are sufficiently misty and obscure to the reader, whatever they may be to the writer. Lastly, the "new operation" is nothing more than what may be now not inappropriately termed "the old operation" of M. Guerin and others.

On one point we most cordially agree with Mr. Childs, and only regret that it is not more generally and faithfully attended to, namely, that members of our profession cannot be too "wary in raising hopes that can never be realized, by submitting the patient to an useless, although *simple* operation." (p. 48.)

The literary qualities of the work are on a par with its scientific value. But we think we have discovered a reason for this. The author has purposely husbanded his powers until the close, and has kept the body of the work dull and subdued, in order that the peroration may burst forth with all its splendour. The latter is so exquisite in its way, that we give it without curtailment:

"Before closing my subject, I cannot but express a regret, that, ere this, some establishment has not been instituted for the reception of those unfortunate beings, who, deprived of the means of procuring for themselves a treatment adapted for their peculiar cases, are doomed to pass their lives neglected and uncared for. Goaded by a sense of their unhappy condition, by the taunts and jeers of those whose minds are unpolished by the refining powers of education, they become thoughtful and morose; bearing about them an impenetrable halo of dark and gloomy thoughts, through which no ray of light can pierce, no gleam of hope, to cheer them through the turbulent stream of life. Their society shunned, their opinions slighted, their views cramped, by the oppressive load under which they toil, their minds yield not to the elasticity and buoyancy of youth; they participate not in the enjoyments of society or the cheering anticipations of domestic love. Their life to them is one cold dreary winter; no summer's sun e'er comes to thaw the frigid ice, which firmly clasps their beating hearts. No smiling spring, with joyous buds that bursting into life impart new charms to all around, can e'er remove from them that one, that all-absorbing grief. Friends who in their earlier life had felt some kindred tie of love, have one by one dropped silent to the grave, and they alone are left, without one social tie to cheer or raise their drooping hearts, but pass through life without a hope, unloving and unloved."

Our armies swore terribly in Flanders; but it was nothing to this!

